# lncRNA TRPM2-AS Promotes Colorectal Cancer Progression by Regulating miR-22-3p and FSTL1

**DOI:** 10.1155/2022/1366511

**Published:** 2022-10-11

**Authors:** Yongsheng Gao, Bing Liu, Xin Liu, Yang Gao, Junqi Shan, Yanliang Li

**Affiliations:** ^1^Department of Pathology, Shandong Cancer Hospital and Institute, Shandong First Medical University and Shandong Academy of Medical Sciences, Jinan 250017, China; ^2^Gastroenterology Surgery Department, Shandong Cancer Hospital and Institute, Shandong First Medical University and Shandong Academy of Medical Sciences, Jinan 250017, China

## Abstract

**Background:**

In recent years, long noncoding RNAs (lncRNAs) relate to many biological processes, which affect the progression of tumors. Transient receptor potential melastatin 2 antisense RNA (TRPM2-AS) is reported to play an oncogene-like role in tumors. TRPM2-AS is highly expressed in colorectal cancer (CRC), but the mechanism of TRPM2-AS is still unclear. The regulatory mechanism of TRPM2-AS in the occurrence of CRC was explored, so as to find new markers and therapeutic targets for CRC.

**Methods:**

TRPM2-AS and miR-22-3p expression in CRC cells were measured through reverse-transcription quantitative polymerase chain reaction (RT-qPCR). Then, TRPM2-AS knockdown cell lines were constructed, and cell counting kit-8 (CCK-8), clone formation, wound healing, and invasion assays were used to detect cell malignant behavior. Follistatin-like 1 (FSTL1) protein was detected by western blotting. The interaction between miR-22-3p and TRPM2-AS or FSTL1 was verified by the luciferase reporter and RNA immunoprecipitation (RIP) assay. Subcutaneous xenografts were performed using animal experiments.

**Results:**

TRPM2-AS expression in CRC cells was increased, and miR-22-3p expression was decreased in CRC cells. TRPM2-AS inhibition inhibited cell malignant behavior. miR-22-3p has a targeting relationship with TRPM2-AS and FSTL1. In cells, downregulation of TRPM2-AS expression promoted miR-22-3p and inhibited FSTL1 expression, while mimics inhibited FSTL1 expression. miR-22-3p inhibition or FSTL1 overexpression could offset the inhibition of TRPM2-AS downregulation on CRC cells.

**Conclusions:**

The TRPM2-AS/miR-22-3p/FSTL1 regulation axis could regulate CRC cell malignant behavior, which may provide a new perspective for interpreting the mechanism of CRC development.

## 1. Introduction

CRC is one most common digestive tract malignancy [1]. Its easy recurrence and metastasis often lead to a poor prognosis for patients [2]. Although treatments such as colonoscopy, colectomy, chemotherapy, and immunotherapy are improving, 5-year survival rates remain poor [3]. Diagnosis and treatment of CRC have made great progression, but its pathogenesis remains unclear. Thus, to further explore CRC progression molecular mechanisms is necessary, which can help improve CRC patient treatment, prognosis, and survival rate.

lncRNA is noncoding functional RNA, which plays an important biological role [4]. lncRNA is dysregulated in various human diseases and relates to the progression, metastasis, and drug resistance of tumors [5]. lncRNA is abnormally expressed in CRC, which relates to the occurrence and development of a tumor and affects the prognosis of patients [6]. Therefore, lncRNAs are expected to be used as disease-specific biomarkers or therapeutic targets.

TRPM2-AS has been reported to have potential diagnostic and prognostic value in many malignancies. Studies have shown that TRPM2-AS can promote the malignant phenotype of ovarian cancer (OC) [7], retinoblastoma (RB) [8], gastric cancer (GC) [9], esophageal cancer (EC) [10], and other tumor cells and then promote the development of tumors. TRPM2-AS downregulation inhibits progression and interferes with cisplatin resistance in OC [7]. TRPM2-AS is upregulated in RB, and TRPM2-AS downregulation obviously inhibits the malignant behavior and promotes apoptosis of RB cells [8]. In GC, TRPM2-AS promotes the progression by miR-612/IGF2BP1 and radioresistance by FOXM1 [9]. In EC, tumorigenesis and metastasis are promoted by TRPM2-AS upregulation [10]. In CRC, TRPM2-AS promotes the proliferation of cells by enhancing TAFL5-mediated TRPM2 mRNA stability [11]. However, the role of TRPM2-AS is poorly studied in CRC. Therefore, exploring the function and mechanism of TRPM2-AS in CRC will be of great significance for marker screening, molecular diagnosis, and targeted therapy of CRC. The aim of this study is to investigate the expression pattern and regulatory role of TRPM2-AS in CRC.

MicroRNAs (miRNAs) are short endogenous noncoding molecules (with length approximately 19 to 23 nucleotides) that regulate target genes via binding to 3′-UTRs [12]. At present, it is widely believed that miRNAs may act as oncogenes or antioncogene in tumor progression [13]. The competitive endogenous RNA (ceRNA) hypothesis has proved that lncRNAs act as miRNA sponges to regulate target genes of miRNA and thus participating in cancer progression [14]. StarBase online target gene prediction software showed that TRPM2-AS may target miR-22-3p. Upregulation of miR-22-3p observably impedes cell proliferation and promotes apoptosis of CRC [15]. However, whether TRPM2-AS interacts with miR-22-3p to regulate the progression of CRC remains to be further elucidated. Therefore, TRPM2-AS expression in CRC progression and possible mechanisms were explored in this study.

## 2. Material and Methods

### 2.1. Cell Culture

NCM-460 and CRC cells (HCT116, DLD-1, SW480, SW620, and LoVo) were cultured in RPMI 1640 (Hyclone, USA), DMEM (Gibco, USA), or McCoy's 5A medium (Gibco) containing 10% fetal bovine serum (FBS) (Gibco), respectively. The culture medium contained 1% double antibody (100 *μ*g/mL streptomycin + 100 U/mL penicillin) in an incubator at 37°C with 5% CO_2_. The cells at the logarithmic growth stage were collected for subsequent experiments.

### 2.2. Cell Transfection

Cells were inoculated into 6-well plates and cultured until cell fusion was about 80%. TRPM2-AS knockdown plasmid (si-TRPM2-AS), miR-22-3p mimic (mimic) and inhibitor (inhibitor), FSTL1 overexpression plasmid (pc-FSTL1), and their negative controls were purchased from GenePharma (Shanghai, China). Transfection was performed according to Lipofectamine 2000 instructions (Invitrogen, USA). After 6 h, cells were replaced with a complete medium and continued for 24 h.

### 2.3. CCK-8 Method

CCK-8 was conducted as previously described [16]. Cells were inoculated into 96-well plates. The plates were placed in an incubator at 37°C with 5% CO_2_ for 24, 48, and 72 h. 10 *μ*L CCK-8 solution was added to each well. The absorbance (*A*) of each well at 450 nm was measured by a microplate reader.

### 2.4. Clone Formation Assay

The colony formation assay was conducted as previously described [16]. Cells were seeded into 6-well plates with 1 × 10^3^ cells/well. The cells were cultured at 37°C for 14 days, fixed with paraformaldehyde, and stained with crystal violet. The clone formation with more than 50 cells was calculated.

### 2.5. Wound Healing Assay

Based on the published reports [17], cells were inoculated in 6-well culture plates. When cells grew to the degree of 80%~90% integration, a fine line was drawn perpendicular to the cells with a sterile head as far as possible. Cells were cleaned with phosphate-buffered saline (PBS) to remove floating cells. The culture medium containing FBS was added at 37°C and cultured for 48 h in a 5% CO_2_ incubator. Scratch width which represented migration ability was measured under a microscope.

### 2.6. Transwell Assay

On the bases of the previously described [18], cells were added to the Transwell upper chamber pretreated with Matrigel (Invitrogen) with 2 × 10^4^ cells/well. 600 *μ*L medium containing 10% FBS was added into the Transwell chamber. After 24 h of culture at 37°C and 5%CO_2_, cells at the bottom of the chamber were fixed and stained. Cells were photographed and counted with the microscope and statistically analyzed.

### 2.7. Luciferase Reporter Assay

The dual-luciferase reporter assay was performed as previously described [19]. Possible TRPM2-AS target miRNAs were predicted by the StarBase online software tool (http://starbase.sysu.edu.cn/), and miR-22-3p target genes were predicted by TargetScan 7.2 (http://www.targetscan.org/vert_72/). Wt (luciferase reporter vector containing TRPM2-AS or FSTL1 binding site) or mut (luciferase reporter vector containing TRPM2-AS or FSTL1 binding site after mutation) and mimics or miR-NC were cotransfected in CRC cells, respectively. Luciferase activity was detected in each group after 48 h culture.

### 2.8. RIP Assay

Following previously described methods [20], the Magna RIP kit (Millipore, USA) was used in the RIP assay according to the instructions. Cells were collected and lysed with RIP lysis buffer. Ago2 immunoprecipitation was then performed using anti-coated anti-human Ago2 antibody magnetic beads, while IgG antibody was used as control. Then, immunoprecipitated RNA was isolated, and expression of TRPM2-AS and miR-22-3p was detected by qRT-PCR.

### 2.9. qRT-PCR

Total RNA was extracted with TRIzol (Invitrogen). After concentration determination, cells were reversed into cDNA using a reverse transcription kit (Takara, China). qRT-PCR was performed by PrimeScript RT Master Mix (Takara) on the Applied Biosystems 7500 real-time PCR system (ABI, USA). RNA relative expression was analyzed by the alternative 2^-∆∆Ct^ method [21].

### 2.10. Western Blot

As described in other articles [22, 23], total protein was extracted by RIPA solution (Beyotime, China) containing protease inhibitor (Roche). Protein sample concentration was detected by a bicinchoninic acid (BCA) protein detection kit (Beyotime). Protein extracts were separated by 10% sodium dodecyl sulfate polyacrylamide gel electrophoresis (SDS-PAGE) and transferred to a polyvinylidene fluoride (PVDF) membrane. The membrane was isolated in 5% skim milk powder for 1 h. The membrane was incubated with the primary antibody (Abcam Company) at 4°C overnight and secondary antibody at room temperature. Electrochemiluminescence solution was added, and images were collected and analyzed in the Bio-rad imaging system.

### 2.11. Tumor Xenograft Model

To evaluate the in vivo tumor growth abilities, the method described before was performed [7]. Cells in each group were prepared into suspension with 1 × 10^6^ cells/mL. Ten female nude mice were randomly divided into 2 groups (5 mice in each group). 0.5 mL cells transfected with sh-NC and sh-TRPM2-AS were injected subcutaneously into the right hind limb of each nude mouse, respectively. After 35 days, nude mice were sacrificed. The transplanted tumor was stripped off and weighed. Tumor volume was calculated as 1/2*ab*^2^ (long diameter (*a*), short diameter (*b*)).

### 2.12. Statistical Analysis

GraphPad Prism 6 software performed the statistical analysis. Data were expressed as $\bar{\text{x}}\pm \text{s}$ and the *t*-test was used to compare the differences between the two groups. *P* < 0.05 was considered statistically significant.

## 3. Results

### 3.1. TRPM2-AS Expression in CRC Cells

**Figure 1:**
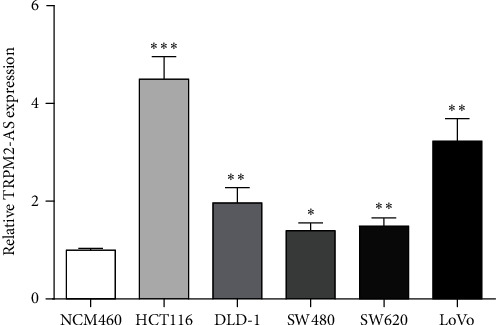
Expression of TRPM2-AS in CRC cells was increased. ^∗^*P* < 0.05,  ^∗∗^*P* < 0.01, and^∗∗∗^*P* < 0.001, compared with NCM460.

TRPM2-AS expression in CRC cells and NCM460 was detected by qRT-PCR. TRPM2-AS expression was found to be increased in CRC cells versus NCM460 (Figure 1). HCT116 and LoVo cells with high TRPM2-AS expression were selected for the next experiment.

### 3.2. Si-TRPM2-AS Inhibits CRC Cell Proliferation In Vitro

**Figure 2:**
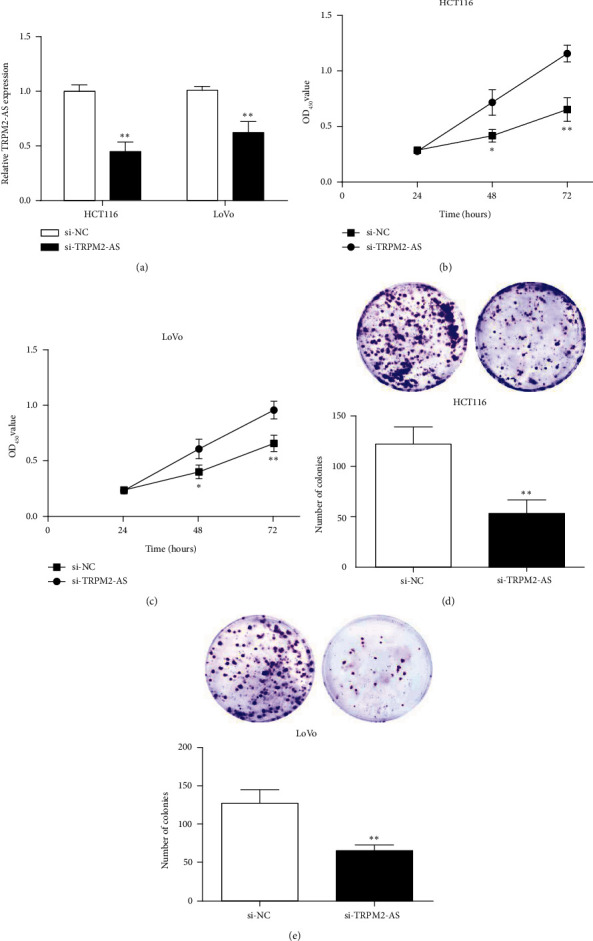
TRPM2-AS inhibition inhibited the proliferation of CRC cells. (a) TRPM2-AS expression was declined in CRC cells transfected with si-TRPM2-AS. (b, c) CCK-8 assay results showed TRPM2-AS inhibition hampered the proliferation activity of CRC cells. (c, d) TRPM2-AS inhibition repressed the clone formation of CRC cells. ^∗^*P* < 0.05,  ^∗∗^*P* < 0.01, compared with the si-NC group.

TRPM2-AS expression was decreased in the si-TRPM2-AS group versus the si-NC group (Figure 2(a)). This suggested that TRPM2-AS was successfully inhibited in HCT116 and LoVo cells.

After transfection, cell proliferation was measured through CCK-8 and clone formation. The proliferation activity of CRC cells was decreased in the si-TRPM2-AS group versus the si-NC group (Figures 2(b) and 2(c)). The clone formation assay showed that after TRPM2-AS inhibition, the number of clones of the two CRC cells decreased versus the control group (Figures 2(d) and 2(e)). This indicated that TRPM2-AS inhibition could reduce CRC cell proliferation.

### 3.3. Effect of TRPM2-AS Inhibition on CRC Cell Migration and Invasion

**Figure 3:**
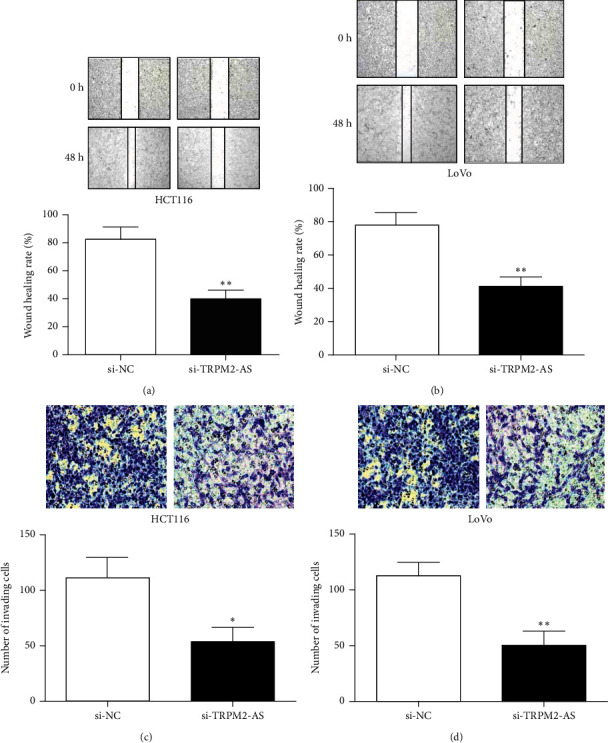
TRPM2-AS inhibition reduced the migration (a, b) and invasion (c, d) ability of GC cells. ^∗^*P* < 0.05,  ^∗∗^*P* < 0.01, compared with the si-NC group.

In CRC cells, the scratch healing ability of the si-TRPM2-AS group was reduced versus the si-NC group at 48 h (Figures 3(a) and 3(b)). The Transwell assay showed that the invading cell number was notably reduced in the si-TRPM2-AS group compared with the si-NC group (Figures 3(c) and 3(d)). These results suggested that TRPM2-AS inhibition could inhibit CRC cell migration and invasion ability in vitro.

### 3.4. TRPM2-AS Targets miR-22-3p

**Figure 4:**
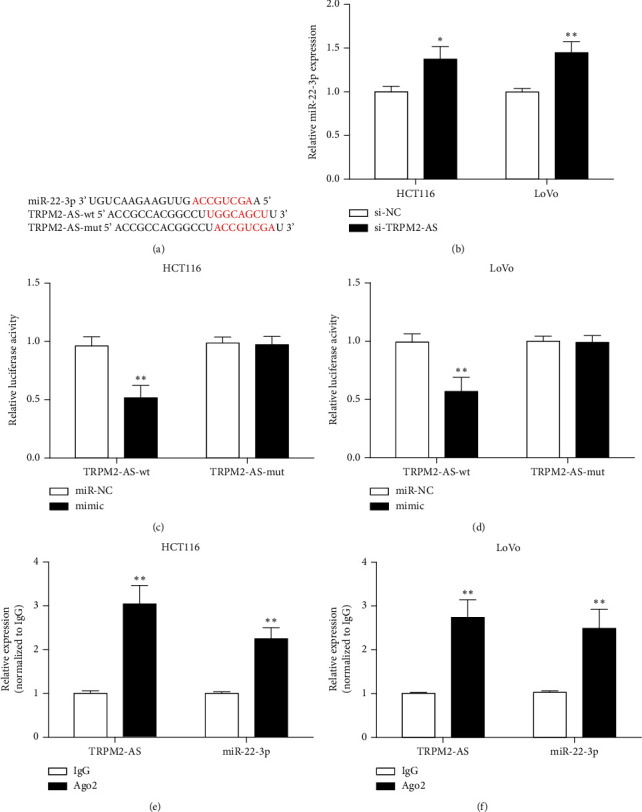
The relationship between TRPM2-AS and miR-22-3p. (a) Bioinformatics software predicted binding sites between TRPM2-AS and miR-22-3p. (b) The expression of miR-22-3p was increased in CRC cells transfected with si-TRPM2-AS. (c, d) Luciferase activity assay determined the relationship between TRPM2-AS and miR-22-3p. (e, f) TRPM2-AS and miR-22-3p expressions in RIP assay. ^∗^*P* < 0.05,  ^∗∗^*P* < 0.01, compared with si-NC, mimic-NC, or IgG group.

StarBase online prediction software showed that TRPM2-AS had targeted binding sites with miR-22-3p (Figure 4(a)). miR-22-3p expression in the si-TRPM2-AS group was increased versus the si-NC group (Figure 4(b)). Luciferase activity was decreased in cells cotransfected miR-22-3p mimic and TRPM2-AS-wt, while luciferase activity has no significant change in other groups (Figures 4(c) and 4(d)). Meanwhile, RIP experiment showed miR-22-3p was enriched in the Ago2 group (Figures 4(e) and 4(f)). These results suggest that TRPM2-AS can target miR-22-3p.

### 3.5. miR-22-3p Targets FSTL1

**Figure 5:**
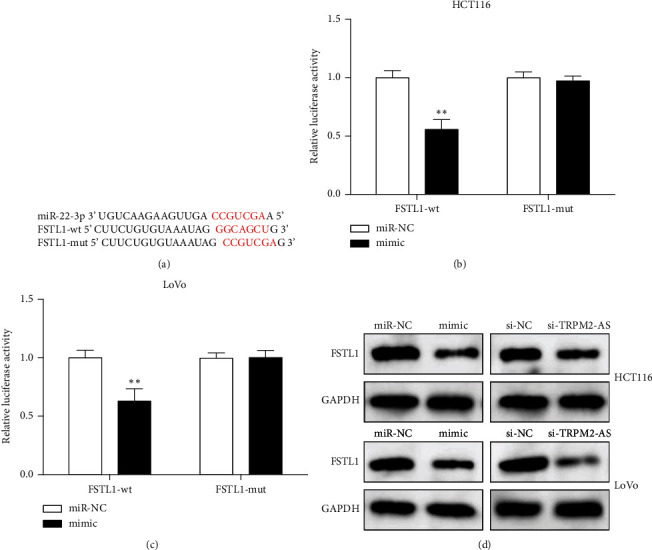
miR-22-3p regulates FSTL1 expression. (a) The binding site between miR-22-3p and FSTL1. (b, c) Dual-luciferase reporter gene assay verified the binding of miR-33-3p and FSTL1. (c) FSTL1 expression was inhibited by miR-22-3p mimic or si-TRPM2-AS. ^∗∗^*P* < 0.01, compared with the miR-NC group.

Bioinformatics software predicted that the miR-22-3p targets FSTL1 (Figure 5(a)). The relative luciferase activity of FSTL1-wt+mimic was declined versus FSTL1-wt and miR-NC cotransfection (Figures 5(b) and 5(c)). WB showed that the FSTL1 protein expression level was obviously decreased after miR-22-3p overexpression or TRPM2-AS inhibition (Figure 5(d)). These suggest that miR-22-3p targets FSTL1.

### 3.6. FSTL1 Overexpression or miR-22-3p Inhibition Partially Offset Effect of TRPM2-AS Low Expression on CRC Cell Malignant Behavior

**Figure 6:**
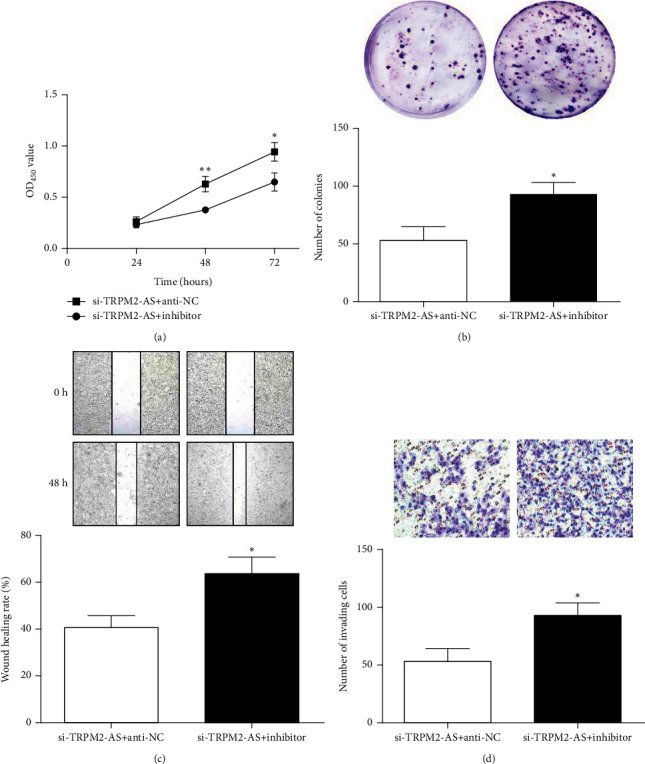
miR-22-3p inhibitor reversed the inhibitory effect of si-TRPM2-AS on proliferation (a, b), migration (c), and invasion (d) of CRC cells. ^∗^*P* < 0.05,  ^∗∗^*P* < 0.01, compared with the si-TRPM2-AS+anti-NC group.

**Figure 7:**
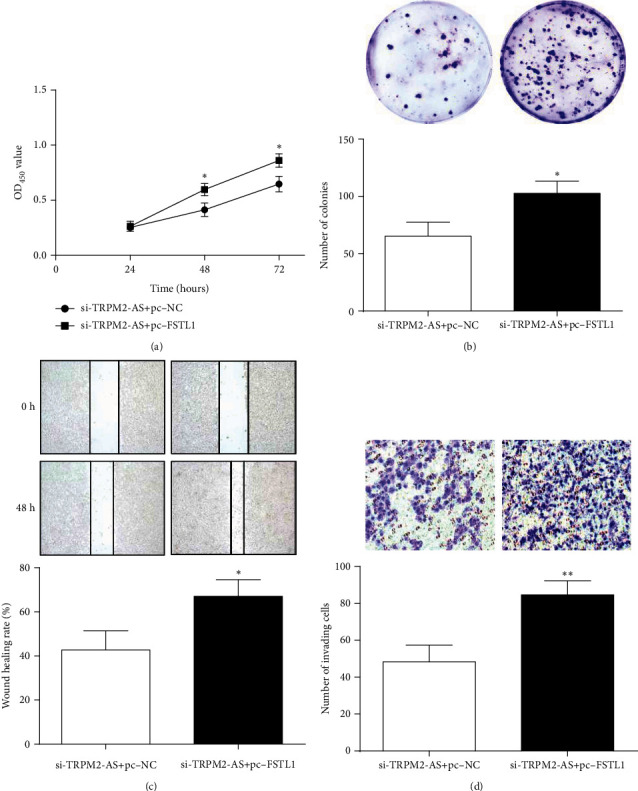
FSTL1 overexpression offset the inhibitory effect of si-TRPM2-AS on proliferation (a, b), migration (c), and invasion (d) of CRC cells. ^∗^*P* < 0.05,  ^∗∗^*P* < 0.01, compared with the si-TRPM2-AS+pc-NC group.

In order to confirm that TRPM2-AS affects CRC cell malignant behavior by miR-22-3p/FSTL1, a rescue assay of FSTL1 overexpression or miR-22-3p inhibition was performed. Compared with the si-TRPM2-AS+anti-NC group, cotransfection of si-TRPM2-AS and inhibitor obviously increased cell viability, colony forming ability, migration ability, and cell invasion number (Figure 6). Similarly, FSTL1 overexpression has the same effect as miR-22-3p inhibition on CRC cells (Figure 7).

### 3.7. TRPM2-AS Inhibition Reduces Tumor Growth in Xenograft Model

**Figure 8:**
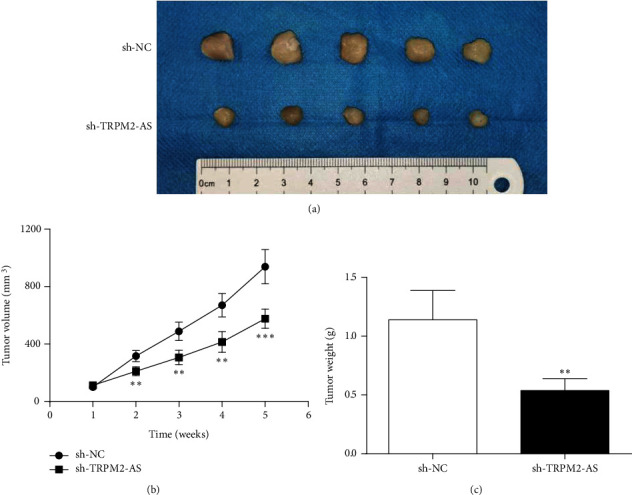
TRPM2-AS knockdown reduces tumor growth in vivo. (a) The xenograft in the sh-NC group was significantly larger than that in the sh-TRPM2-AS group. (b) The volume of xenograft in sh-NC and sh-TRPM2-AS groups. (c) The weight of xenograft in the sh-NC control group was significantly higher than that in the sh-TRPM2-AS group. ^∗∗^*P* < 0.01,  ^∗∗∗^*P* < 0.001, compared with the sh-NC group.

The effect of TRPM2-AS on CRC cell proliferation in vivo was also explored. In our experiment, HCT116 cells transfected with short hairpin RNAs for TRPM2-AS (sh-TRPM2-AS) or its control (sh-NC) were selected for tumor formation by subcutaneous injection. Tumor tissue in the sh-TRPM2-AS group was smaller than that in the sh-NC group (Figure 8(a)). Tumor volume has the same trend as tumor tissue (Figure 8(b)). The tumor weight of the sh-TRPM2-AS group was markedly decreased versus the sh-NC group (Figure 8(c)). Thus, TRPM2-AS inhibition could significantly hamper the proliferation of CRC cells in vivo.

## 4. Discussion

Most CRC patients are diagnosed as advanced and miss the chance of radical surgery [24]. Early diagnosis and early treatment can effectively reduce the mortality of CRC patients and improve the cure rate. With the development of precision medicine, new biomarkers should be developed to help patients' diagnosis, improve the treatment effect, improve the prognosis, and better guide clinical practice. In CRC, many studies reported that lncRNA is abnormally expressed, which has potential application value in the diagnosis, prognosis assessment, drug resistance assessment, targeted therapy, and other aspects of CRC [25].

lncRNA is a multifunctional noncoding regulatory transcript. Researches on the relationship between differentially expressed lncRNAs and the mechanism of tumorigenesis and development are also being gradually carried out and deepened. TRPM2-AS is a newly discovered noncoding RNA molecule that promotes cancer progression in RB, GC, EC, CRC, and other cancers [7–10]. We found that TRPM2-AS expression was increased in CRC cells and the inhibition of TRPM2-AS hampered CRC cell proliferation, which was consistent with a previous study [11]. Moreover, the scratch healing ability of cells and the number of invading cells were notably decreased by TRPM2-AS inhibition. This suggests that TRPM2-AS downregulation inhibits CRC cell proliferation, migration, and invasion.

lncRNAs can act as miRNA precursors to regulate mRNA stability and have great potential in tumor diagnosis and treatment [26]. In order to further analyze the mechanism of TRPM2-AS in the occurrence and development of CRC, StarBase software was used to predict the microRNA molecules that TRPM2-AS might bind. It was found that miR-22-3p could bind TRPM2-AS. Tian et al. suggested that bladder cancer progression could be promoted by TRPM2-AS through miR-22-3p and GINS2 [19]. LINC00858 promoted progression by the miR-22-3p/YWHAZ axis in CRC [27]. miR-22-3p expression was decreased in CRC, and its overexpression hampered CRC cell malignant behavior [28]. RIP and double luciferase experiment in this study showed that TRPM2-AS could complement miR-22-3p. miR-22-3p expression was increased by TRPM2-AS inhibition, suggesting that TRPM2-AS may negatively regulate miR-22-3p in CRC.

Furthermore, TargetScanHuman 7.2 website prediction showed that miR-22-3p may complement FSTL1 mRNA. FSTL1, an extracellular glycoprotein, is associated with cell survival, proliferation, differentiation and migration, and embryonic organ maturation [29]. FSTL1 not only can be used as a marker for disease diagnosis and development but also relates to the occurrence and development of related diseases [29]. FSTL1 is upregulated in many solid tumor cells including glioma, gastric cancer, and hepatocellular carcinoma, and inhibiting the expression of FSTL1 can effectively reduce various malignant biological behaviors of cancer cells [30–32]. In CRC, FSTL1 is upregulated, and FSTL1 stable overexpression can promote CRC cell migration and invasion and shorten the survival time of nude mice [33–35]. This study confirmed that miR-22-3p targets FSTL1. What is more, both TRPM2-AS inhibition and miR-22-3p overexpression markedly decreased FSTL1 expression. Moreover, rescue further experiments showed that the effect of TRPM2-AS inhibition of CRC cell malignant behaviors was partially relieved after miR-22-3p inhibition or FSTL1 overexpression. These results indicated that the TRPM2-AS/miR-22-3p/FSTL1 signaling axis may be involved in regulating CRC progression. This study not only has a certain reference value for the study of the mechanism of CRC metastasis but also has important significance for the study of its treatment mechanism. However, the current research content of this study is not in-depth and needs to be further verified in a later stage, such as TRPM2-AS expression in the tissues of patients with CRC and its relationship with clinicopathological characteristics and the effect of TRPM2-AS on CRC cell apoptosis.

## 5. Conclusion

This study demonstrated that TRPM2-AS was significantly increased in CRC. Mechanistically, TRPM2-AS could regulate CRC malignant behavior by inhibiting the miR-22-3p/FSTL1 axis. This proves the role of the TRPM2-AS/miR-22-3p/FSTL1 molecular axis in CRC and provides an experimental basis for the diagnosis and targeted therapy of CRC.

## Data Availability

The datasets used and/or analyzed during the present study are available from the corresponding author on reasonable request.
